# Changes in Blood Components in Aphtha Patients with Excess Heat

**DOI:** 10.1155/2016/7219257

**Published:** 2016-06-08

**Authors:** Lu Qin, Yan Li, Yifeng Jiao, Danqing Fu, Li Ye, Jinjun Ji, Guanqun Xie, Yongsheng Fan, Li Xu

**Affiliations:** College of Basic Medicine, Zhejiang Chinese Medical University, Hangzhou, Zhejiang 310053, China

## Abstract

“Superior heat” is a popularization expression in TCM heat syndrome and has no counterpart in the modern medical system concept. Oral ulcer is considered to be a kind of clinical manifestation of “superior heat.” Aphtha is a common and frequently occurring disease, which can be divided into excess heat and Yin deficiency. The aphtha of excess heat manifests the syndromes of acute occurrence, severe local symptoms, obvious swelling and pain, red tongue, yellow coating, and fast-powerful pulse. In this study, we found that there was an abnormal immune regulation in aphtha patients induced by excess heat. There are changes in the blood components, including abnormal serum protein expression (IL-4, MMP-19, MMP-9, and Activin A) and a higher percentage of CD4^+^CD25^+^Treg cells in the peripheral blood lymphocytes of the EXP group. Changes in the blood environment may be an important factor in the occurrence of aphtha caused by excess heat.

## 1. Introduction

“Superior heat” is often used to describe mouth sores, sore throat and gums, and other physical symptoms. Among people familiar with the term “superior heat,” originating from traditional Chinese culture and TCM, it is a popularized expression referring to TCM heat syndrome and has no counterpart concept in the modern medical system. The oral ulcer is considered to be a type of clinical manifestation of “superior heat.” Aphtha, also known as “Kou Gan,” “Kou Yang,” “Kou mi,” and “Kou po” in the medical works of various dynasties [1], is a recurrent mouth mucosa ulcer with a characteristic of yellowish-white ulcers around the gum, tongue, cheeks, and palate, with pain, salivation, and sometimes fever. The ulcer, with an obvious feeling of burning pain and an ability to heal in 7–10 days, is usually round or oval and can be single or multiple [2, 3]. Aphtha is a common and frequently occurring disease that can be divided into excess heat and yin deficiency [4]. The aphtha of excess heat is characterized by acute occurrence, severe local symptoms, obvious swelling and pain, red tongue, yellow coating, and a fast and powerful pulse. It is occasionally combined with flushing, hot eyes, dry and cracked lips, a bitter taste in the mouth, a dry and thirsty mouth, aphthous stomatitis, swollen sore throat, bleeding gums, epistaxis, sores and furuncles, fever or aversion to heat, dysphoria, dark urine, constipation, and other symptoms. The aphtha of yin deficiency is characterized by internal heat due to yin deficiency as a long course, mild local symptoms, repeated attacks, mild swelling and pain, a red tongue with little fluid, scanty coating or no coating, and a thin and fast pulse, sometimes combined with hot flashes, night sweat, emaciation, dry throat and mouth, dysphoria with feverish sensation in the chest, palms, and soles, and other symptoms [5]. This type of syndrome classification provides the basis for clinical treatment. The clinical application of herbs for the aphtha of excess heat mainly involves radix scutellariae (黄芩), rhizoma coptidis (黄连), fructus forsythia (*连翘*), folium isatidis (大*青叶*), and other herbs, while the treatment of mouth ulcers due to yin deficiency mainly involves radix rehmanniae (生地), herba asari (细辛), radix ophiopogonis (麦冬), cortex phellodendri (黄柏), cortex cinnamomi (*肉桂*), and other herbs [6]. This paper aims to investigate the immune regulation mechanism of aphtha caused by excess heat from the perspective of molecular immunity.

The protein chip is a tool for the high-throughput expression profiling of protein function. The antibody detection chip [7], a type of protein chip, has the antibody fixed on the surface of the solid phase carrier and captures the antigen in the sample through a specific immune response. It can detect the expression abundance of hundreds of proteins simultaneously. By applying the protein chip to research the different presentation of immune regulation-related cytokines in the serum of patients suffering from aphtha of excess heat, we discuss the changes to immune homeostasis characteristics in this type of aphtha and elucidate its immune regulation-related mechanism. The antibody chip results showed that indices such as Activin RII A/B, BLC/BCA-1/CXCL13, and Cryptic exhibit significant and significant differences. Based on the chip results, we chose a series of serum proteins for further testing by ELISA in more specimens. The results showed a significant difference between the two groups. Changes in the factors in the serum of the patients suffering from aphtha enriched the scientific information on aphtha of excess heat and provided new ideas for the clinical treatment and mechanism of drug action. Treg cells are a category of cells with immunosuppressive effects on subsets of T cell. They are involved in the fine regulation of immune response or immune tolerance and can inhibit autoimmune effects and protect tissue from rejection injury, and they have an important role in the reduction of autoimmunity and in transplantation [8]. Studies have found that Treg cells are closely related to mucosal immunity [9, 10]. In this study, the flow cytometry results showed that the percentage of CD4^+^CD25^+^Treg cells in the peripheral blood lymphocytes of the EXP group was higher than in the NOR group.

## 2. Materials and Methods

### 2.1. Patients

Six patients diagnosed with aphtha due to excess heat according to TCM syndrome were regarded as the EXP group. When these 6 patients fully recovered, they were used as the self-control group (CON group). Subsequently, 20 patients with TCM syndrome of the excess heat type “superior heat” were recruited as the experimental group (EXP group), and 20 healthy people whose TCM syndrome was of the yin-yang harmony type were recruited as the normal group (NOR group).

### 2.2. Blood Collection and Serum Preparation

First, 5 mL of fasting venous blood samples was collected from each participant in the morning in EDTA tubes and clotting tubes. In brief, the clotting tubes were placed at room temperature for 2 hours and then centrifuged at 1000 g for 15 min. The supernatant (i.e., serum) was collected and stored at −80°C. Repeated freeze-thaw cycles were avoided.

### 2.3. Extraction of Human Blood Lymphocytes

The blood of the EXP group and NOR group was centrifuged using the peripheral blood lymphocyte separation liquid at 1000 g for 15 min. The peripheral blood lymphocytes layer was obtained, and the middle layer of white floc was drawn gently and washed once with RPMI-1640 high glucose medium. The counting and cell concentration were adjusted for 1 × 10^7^ mL^−1^, in preparation for flow cytometry.

### 2.4. Reagents

RayBio L-series Human Antibody Array L-507 Slides (No. AAH-BLG-1-4) were purchased from RayBiotech Company. The IL-4 ELISA test kit (KE1039) was purchased from Immunoway Company in the United States. The MMP-19 (XF01632B) ELISA test kit was purchased from Xin Fan Biotech Co., Ltd., in Shanghai. The Activin A (EK0301) ELISA test kit and MMP-9 (EK0465) ELISA test kit were purchased from Boster Biological Co., Ltd. Human peripheral blood lymphocyte separating liquid (No. LTS1077) was purchased from Tianjin Hanyang Biologicals Co., Ltd. BD Pharmingen*™* FITC Mouse Anti-Human CD4 (No. 555346) and BD Pharmingen PE Mouse Anti-Human CD25 (No. 555432) were purchased from BD Biosciences.

### 2.5. Protein Chip

A wide array of 507 proteins (including cytokines, chemokines, adipokines, growth factors, angiogenic factors, proteases, soluble receptors, and soluble adhesion molecules) were detected using the with Human Cytokine Antibody Array Kit according to the manufacturer's instructions. Briefly, the operation was conducted according to the standard procedure of the RayBiotech kit. First, the sample proteins were labeled with biotin prior to incubation with the capture antibodies, followed by dialysis to remove free biotin. From here, the newly biotinylated sample was placed on the array membrane and incubated at room temperature, washed, and then incubated with Cy3-conjugated-streptavidin. The signals were visualized by fluorescence. Finally, the relative expression levels of the proteins were quantified by densitometry.

### 2.6. ELISA

The experimental method was performed according to the instructions for the ELISA kits. In brief, 100 *μ*L of the appropriately diluted sample was added to each well and incubated for 90 min at 37°C. Subsequently, the samples were removed, and the plate was washed twice. Next 100 *μ*L of detection antibody was added to each well and incubated for 2 hours at room temperature. The plate was washed four times with PBS, and then 100 *μ*L of HRP-conjugated secondary antibody was added followed by incubation for 1-2 hours at room temperature. The plate was then washed four times with PBS. Finally, the optical densities of IL-4, MMP-19, MMP-9, and Activin A were measured by a microplate reader in a multiwavelength measurement system (Thermo Scientific Varioskan Flash).

### 2.7. Flow Cytometry

For this purpose, 100 *μ*L of samples of peripheral blood lymphocyte suspension “superior heat” patients and normal healthy people was added to the flow tube, with 5 *μ*L of FITC-Anti-Human CD4 and PE-Anti-Human CD25, respectively, mixed fully, then kept from light and incubated for 30 min at room temperature, and centrifuged at 900 g for 10 min. The supernatant was removed, and the cells in each group were resuspended in 500 *μ*L of FBS. The percentage changes of CD4^+^CD25^+^Treg cells in the NOR group and EXP group were detected by flow cytometry.

### 2.8. Statistical Analysis

All values were expressed as the mean ± standard deviation. The independent-samples *t*-test was used for comparisons between two groups if the variance was orderly. Comparison between 2 groups was performed using a nonparametric test if the variance was not orderly. Differences were considered statistically significant if the *P* value was less than 0.05. Analyses were performed using SPSS version 16.0 (SPSS Inc., Chicago, IL, USA).

## 3. Results

### 3.1. Hierarchical Clustering Analysis of Serum Protein Expression

**Figure 1:**
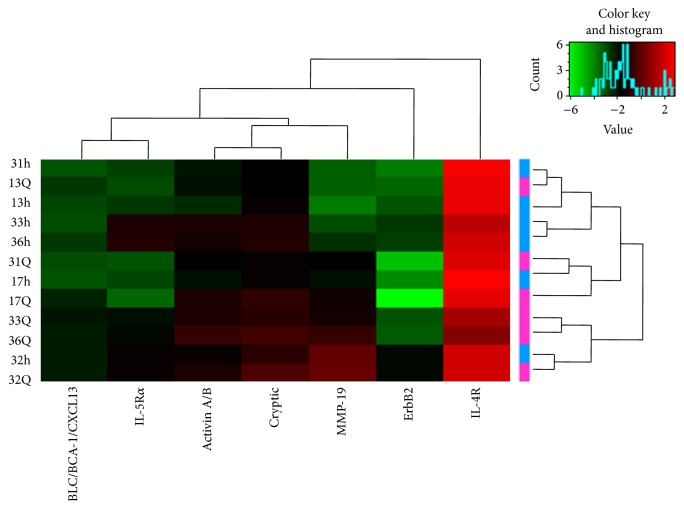
Cluster analysis of significantly different serum protein expression. Heat map generated from protein microarray data reflecting protein expression values of the 7 proteins in all enrolled participants. Each row represents a sample, and each column represents an antibody. The antibody clustering tree is shown at the top. The color scale shown in the upper right corner illustrates the relative expression level of an antibody in the slide: red represents a high relative expression level, and green represents a low relative expression level.

Two major clusters were identified by clustering analysis based on 507 types of serum proteins expressed in the serum of 6 EXP patients (13Q, 17Q, 31Q, 32Q, 33Q, and 36Q) and 6 CON patients (13 h, 17 h, 31 h, 32 h, 33 h, and 36 h). The RayBio L-series Human Antibody Array L-507 Glass Slides chip figure was extracted by scanning the chip data, and after correct treatment, the expression differences of 507 types of proteins between the two groups were analyzed by software. A total of 34 proteins were screened for differential expression. The expression levels of 7 proteins were significantly different (Figure 1).

### 3.2. Volcano Plot of Differentially Expressed Serum Protein

**Figure 2:**
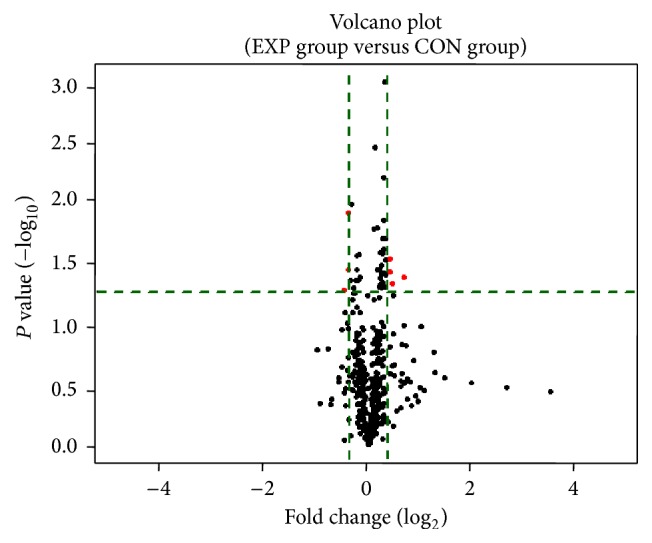
Volcano plot of differentially expressed serum protein. The upper part of the green dotted line represents the 34 proteins with statistically significant differential expression. The left three red dots represent proteins with differential expression downregulated by 1.3-fold in the EXP group. The right four red dots represent proteins with differential expression upregulated by 1.3-fold in the EXP group.

The volcano plot analyzes 507 types of serum proteins for differences in expression between the EXP group and CON group. The log_2_ (fold change) value of the differential protein expression is taken as the abscissa, and the negative logarithm-log_10_ (*P* value) of the significance test *P* values between the two groups (EXP group and CON group) is taken as the ordinate. Red dots represent the proteins exhibiting upregulated or downregulated differential expression with a difference of 1.3 fold in the EXP group, whose differential expression has statistical significance (Figure 2).

### 3.3. Analysis of 7 Significantly Differentially Expressed Serum Proteins between the EXP Group and CON Group

Compared with the 6 people in the CON group, the average expression levels of 4 types of proteins (ActivinRII A/B, BLC/BCA-1/CXCL13, Cryptic, and MMP-19) increased by 1.3-fold in the serum of the 6 people in the EXP group (*P* < 0.05), and the expression of 3 types of proteins (ErbB2, IL-4R, and IL-5R alpha) in the serum of EXP group decreased by 1.3-fold (*P* < 0.05) (Table 1).

### 3.4. Serum IL-4, MMP-19, MMP-9, and Activin A Levels Analyzed in NOR Group and EXP Group

**Figure 3:**
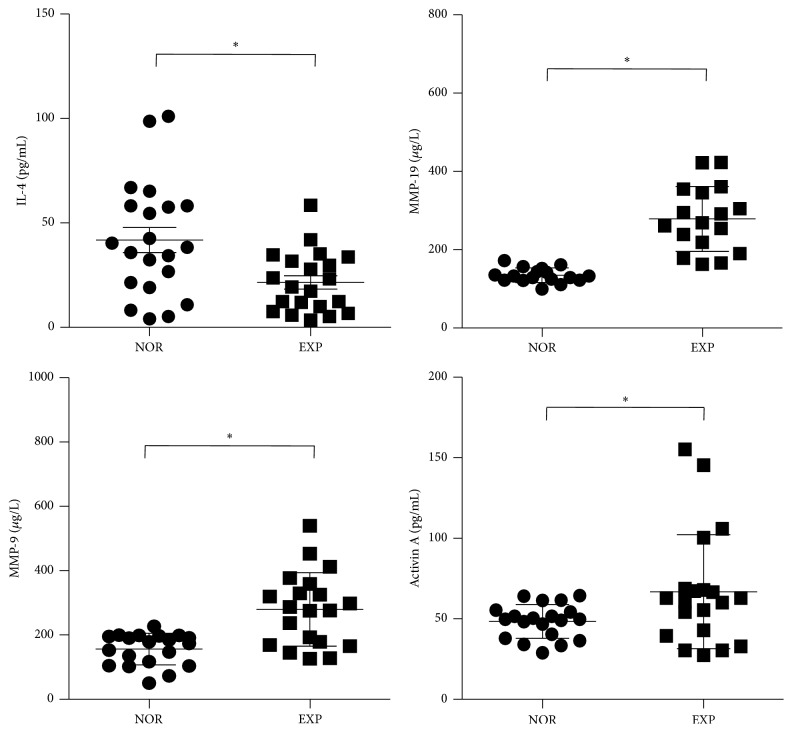
IL-4, MMP-19, MMP-9, and Activin A levels of serum samples in two groups. Note: NOR: normal group and EXP: experiment group. Comparing with NOR group, ^*∗*^ *P* < 0.05.

The expression levels of IL-4, MMP-19, and Activin A are closely related to the occurrence of oral ulcers. In the latter part of the experiment, we found that MMP-9 was also abnormally expressed in the excess heat period of oral ulcers. To validate the results for IL-4, MMP-19, MMP-9, and Activin A in the larger sample, we collected serum from 40 volunteers for further validation. The ELISA results showed that compared with the NOR group, the level of IL-4 in the serum decreased significantly in the EXP group (*P* < 0.05), but the serum levels of MMP-19, MMP-9, and Activin A increased significantly (*P* < 0.05) (Figure 3).

### 3.5. Percentage of CD4^+^CD25^+^Treg Cells in the Peripheral Blood Lymphocytes from the NOR Group and EXP Group

**Figure 4:**
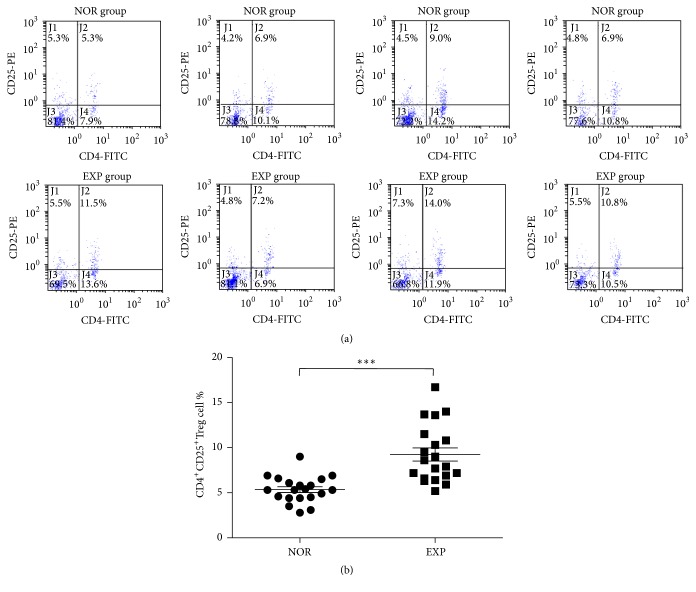
Percentage of CD4^+^CD25^+^Treg cells in the peripheral blood lymphocytes from the NOR group and EXP group. (a) Flow cytometry of CD4^+^CD25^+^Treg cells from partial samples of the NOR group and EXP group. (b) Percentage of CD4^+^CD25^+^Treg cells in the peripheral blood lymphocytes from the NOR group and EXP group (*n* = 20 per group). Note: NOR: normal group; EXP: experiment group. Comparing with the NOR group, ^*∗∗∗*^ *P* < 0.0001.

A double-parameter map was made showing the FITC and PE fluorescence intensity. The cells were divided into four subgroups in the two-dimensional FCM map. The right upper quadrant shows FITC+/PE+ cells for the CD4^+^CD25^+^Treg cells group. Flow cytometry showed that the percentage of CD4^+^CD25^+^Treg cells in the peripheral blood lymphocytes of the EXP group was higher than in the NOR group, and the difference was statistically significant (*P* < 0.0001) (Figure 4).

## 4. Discussion

Aphtha of excess heat is a common and frequently occurring disease, similar to recurrent oral ulcer, gingivitis, and periodontitis. Balancing the human immune system plays an important role in oral immune defense [11]. The RayBiotech biotin labeled antibody chip, which can detect the expression levels of 507 types of human serum proteins including cytokines, was selected to detect the serum protein expression of aphtha patients with excess heat. The results showed a variety of differential expression proteins in aphtha patients, including four types of proteins with significant rising and three types of proteins with significant reduction (Table 1). BLC/BCA-1/CXCL13 is a small cytokine belonging to the CXC chemokine family [12]. It preferentially promotes the migration of B lymphocytes, apparently by stimulating calcium influx into cells expressing Burkitt's lymphoma receptor 1 (BLR-1), and functions in the homing of B lymphocytes to lymphoid follicles [13]. This research showed that the expression of serum CXCL13 in the EXP group was upregulated, which enabled the B lymphocyte to mediate the humoral immune protection mechanism. Cryptic is a member of the epidermal growth factor (EGF-) CFC (EGF-CFC) family, which has similar structure to the EGF-CFC protein family. Studies showed that the EGF-CFC protein played an important role in promoting cell proliferation and migration [14]. Regarding the increased Activin RII A/B expression in the EXP group, we also found increased Activin A and Activin B, which combined with Activin RII A/B receptor to function. Because Activin A and Activin B increased by 1.28-fold and 1.19-fold in the EXP group, respectively, the data are not shown. Activin was highly expressed in injured skin [15, 16]. The increased Activin A/B and Activin RII A/B expression may have a protective effect on damaged oral mucosa.

In addition to the increased proteins, 3 types of proteins (ErbB2, IL-4R, and IL-5R alpha) in the serum of EXP group decreased by more than 1.3-fold. The IL-5R*α* chain is exclusively expressed by eosinophils. The soluble form does not lead to signal transduction and therefore has an antagonistic effect on IL-5 signaling [17].

Matrix metalloproteinases (MMPs) are a type of endopeptidase that play a central role in cell proliferation, migration, differentiation, and host defences [18]. MMP-19 was firstly found in the synovial membrane of rheumatoid patients. T cell-mediated immune response is impaired in MMP-19 knockout mice with skin allergic reactions, which showed that MMP-19 plays an important part in skin immune response [19]. MMP-9 also plays an important role in immune cell activity [20]. Our results showed increased MMP-9 and MMP-19 in the EXP group, which indicated that MMP-9 and MMP-19 were related to the process of aphtha of excess heat by regulating the immune response.

IL-4 can make IgM type B cells into IgA type B cells and promote increases in the number of secretory IgA cells [21]. It also exhibited a certain effect on the recovery of intestinal mucosal immune function after radiation [22]. Some scholars have reported that IL-4 can facilitate the secretion of IgA receptor-SC by epithelial cell lines (HT29) through activating tyrosine protein kinase and then forming sIgA, so IL-4 plays an important role in the formation of sIgA [23]. This experiment showed that the abnormal expression of IL-4 may be closely related to the occurrence and development of excess heat type aphtha.

CD4^+^CD25^+^Treg cells play an important role in maintaining immune homeostasis and immune tolerance at the cellular level [9, 24]. It was found that the percentage of peripheral blood lymphocytes in the EXP group was higher than in the NOR group in the larger sample, and the difference was statistically significant. We found that Treg cells may be involved in the pathogenesis of the excess heat type “superior heat” and have elevated expression in “superior heat.” As reported in the literature, IL-4 is closely involved in the regulation of Treg cells [25]. In this work, we found that the secretion levels of IL-4R and IL-4 in serum are decreased, while the content of Treg cells is increased. This result needs further analysis to determine whether it occurred because of the higher IL4R in the cell.

In conclusion, our results showed variations in serum proteins, especially MMP-19, MMP-9, IL-4, and Activin A in patients with aphtha caused by the excess heat type “superior heat.” The discovery of these molecular immune targets can provide a reference for finding drugs for the prevention and treatment of excess heat type aphtha. However, the pathogenesis of “superior heat” and the abnormal relationship with Treg cells require further research.

## Figures and Tables

**Table 1:** Seven types of significantly differentially expressed serum proteins.

Different proteins	EXP group	CON group	Fold change	*P* value
Activin RII A/B	0.413 ± 0.127	0.306 ± 0.090	1.35	0.026
BLC/BCA-1/CXCL13	0.195 ± 0.049	0.140 ± 0.038	1.39	0.043
Cryptic	0.521 ± 0.178	0.390 ± 0.082	1.33	0.034
MMP-19	0.476 ± 0.336	0.292 ± 0.369	1.63	0.038
ErbB2	0.105 ± 0.100	0.138 ± 0.084	0.75	0.011
IL-4R	3.826 ± 1.557	5.022 ± 1.733	0.76	0.033
IL-5R alpha	0.197 ± 0.100	0.277 ± 0.149	0.71	0.048
